# Ocular phenotype and genetical analysis in patients with retinopathy of prematurity

**DOI:** 10.1186/s12886-022-02252-x

**Published:** 2022-01-12

**Authors:** Tianchang Tao, Xianfen Meng, Ningda Xu, Jiarui Li, Yong Cheng, Yi Chen, Lvzhen Huang

**Affiliations:** 1 Department of Ophthalmology, Peking University People’s Hospital, Eye Diseases and Optometry Institute, Beijing, China; 2 Beijing Key Laboratory of Diagnosis and Therapy of Retinal and Choroid Diseases, Beijing, China; 3 College of Optometry, Peking University Health Science Center, Beijing, China; 4 Department of Ophthalmology, Beijing Huaxin Hospital, The First Hospital of Tsinghua University, Beijing, China; 5 Department of Ophthalmology, China-Japan Friendship Hospital, Beijing, China

**Keywords:** Retinopathy of prematurity, Frizzled-4, Wnt signaling pathway, Retinal vascular development

## Abstract

**Background:**

Retinopathy of prematurity (ROP) is a multifactorial retinal disease, involving both environmental and genetic factors; The purpose of this study is to evaluate the clinical presentations and genetic variants in Chinese patients with ROP.

**Methods:**

A total of 36 patients diagnosed with ROP were enrolled in this study, their medical and ophthalmic histories were obtained, and comprehensive clinical examinations were performed. Genomic DNA was isolated from peripheral blood of ROP patients, polymerase chain reaction and direct sequencing of the associated pathogenic genes (*FZD4*, *TSPAN12*, and *NDP*) were performed.

**Results:**

All patients exhibited the clinical manifestations of ROP. No mutations were detected in the *TSPAN12* and *NDP* genes in all patients; Interestingly, three novel missense mutations were identified in the *FZD4* gene (p.A2P, p.L79M, and p.Y378C) in four patients, for a detection rate of 11.1% (4/36).

**Conclusions:**

This study expands the genotypic spectrum of *FZD4* gene in ROP patients, and our findings underscore the importance of obtaining molecular analyses and comprehensive health screening for this retinal disease.

**Supplementary Information:**

The online version contains supplementary material available at 10.1186/s12886-022-02252-x.

## Background

Retinopathy of prematurity (ROP) is a complex ocular disease, characterized by abnormal retinal vascularization in premature infants; This disease is firstly described as retrolental fibroplasia in 1940s when a grayish white, opaque membrane behind the lens was observed in preterm infants [1]. ROP has become one of the leading causes of childhood blindness across the world, and the prevalence of ROP varies widely in different countries or areas [2–4], which should be associated with the regional economic development level and public health facilities. There is a two-phase hypothesis of ROP [5], in first phase, delayed retinal vascular development leads to a peripheral avascular area of the retina, and in second phase, angiogenesis can happen at the junction of avascularized and vascularized retina as a result of pathological compensatory mechanism, this process is considered to be attributed to the aberrant changes of oxygen tension and vascular endothelial growth factor (VEGF) level.

Although the exact mechanism of ROP remains unclear, many risk factors have been reported to induce ROP [6–9], such as low birth weight, young gestational age, supplemental oxygen, mechanical ventilation, apnea, anemia, and blood transfusion. In addition to those environmental factors, genetic mutations are also considered to get involved in ROP development, especially Wnt signaling factors: Frizzled-4 (FZD4), Tetraspanin-12 (TSPAN12), and Norrin (NDP), this signaling system plays and important role in fetal vascular formation of retina and inner ear [10, 11], and its functional disruption could be associated with the pathogenesis of some retinal vascular disorders like Coat’s disease and familial exudative vitreoretinopathy (FEVR) [12, 13]. Because of the phenotypic similarities between ROP and FEVR, some studies indicate the possibility of involvement of Wnt signaling genes mutations in the occurrence of ROP [14–16]. Thus, further studies are required to investigate the correlation between Wnt signaling abnormality and aberrant retinal vascular development in ROP patients.

Here, our study investigated 36 patients diagnosed with ROP, we obtained comprehensive histories of their illnesses, and then evaluated the effect of pathogenic genes variants on their ocular features.

## Methods

### Patients

Thirty-six ROP patients from thirty-three unrelated families were enrolled in this study, the informed consent was obtained from all guardians on behalf of the participants. Our study conformed to the tenets of the Declaration of Helsinki. The procedure was approved by the ethics committee of Peking University People’s Hospital.

Data for gender, gestational age (GA) at birth, birth weight (BW) were collected, and complete medical histories were consulted if available. The results of ophthalmic examinations, such as indirect ophthalmoscopy, slit-lamp biomicroscopy, and color fundus photography, were collected where available. The clinical tests obtained in our study were performed before treatment. The stage of ROP was assessed for each patient following the International Classification of Retinopathy of Prematurity [17]: Stage 0 presents only immature retinal vasculature; Stage 1 presents a demarcation line between vascularized and avascular retina; Stage 2 presents a ridge characterized by the demarcation line with height, width, and volume; Stage 3 presents the extraretinal fibrovascular proliferation or neovascularization extending from the ridge into the vitreous; Stage 4 is divided into extrafoveal (4A) or foveal (4B) partial retinal detachment; Stage 5 presents the total retinal detachment.

### Molecular analysis

Genomic DNA was extracted from peripheral blood of ROP patients using an Agilent SureSelect Target Enrichment System Kit (Agilent, USA). Polymerase chain reaction (PCR) was performed using Goldstar Taq MasterMix (Cwbio, PRC) to amplify the exons of the *FZD4*, *TSPAN12*, and *NDP* genes. The samples were sequenced and analyzed by NEXTSEQ500 (Illumina, USA) as described previously [18].

The possible pathogenicity of novel missense mutations would be evaluated by using SIFT (http://sift.jcvi.org/), PolyPhen-2 (http://genetics.bwh.harvard.edu/pph2/), and Mutation Taster (http://www.mutationtaster.org) prediction software and via evolutionary conservation analysis. The minor allele frequencies (MAF) of variants in participants were checked using Exome Aggregation Consortium database (http://exac.broadinstitute.org/faq) and Genome Aggregation database (http://gnomad-sg.org/); All mutations were also evaluated regarding pathogenicity according to the standards and guidelines of American College of Medical Genetics and Genomics (ACMG) [19].

## Results

Demographic data for the 36 participants are presented in Table 1. Twenty-three patients were boys (63.9%) and thirteen were girls (36.1%). The average gestational age was 30 weeks with a range from 27 to 36 weeks, and the average birth weight was 1300 g with a range from 890 to 2700 g; Thirty-three patients (91.7%) presented the ocular manifestation of classic ROP, and the aggressive posterior ROP (AP-ROP) was found in three patients (8.3%); Among the 36 cases, three patients (8.3%) were at stage 0, one patient (2.8%) was at stage 1, one patient (2.8%) was at stage 2, 20 patients (57.4%) were classified as stage 3, two patients (5.6%) were at stage 4A, two patients (5.6%) were at stage 4B, and the other seven patients (19.3%) were not determined. Twenty-eight patients (77.8%) underwent intravitreal anti-VEGF agent or laser photocoagulation after screening and diagnosis.

**Table 1:** Demographic data for 36 patients with retinopathy of prematurity

**Gestational age**	27 – 36 (mean 30) weeks
**Birth weight**	890 – 2700 (mean 1300) grams
**Sex**	Male: 23 (63.9%)
	Female: 13 (36.1%)
**Type of ROP**	AP-ROP: 3 (8.3%)
	Classic ROP: 33 (91.7%)
**Stage of ROP**^**a**^	Stage 0: 3 (8.3%)
	Stage 1: 1 (2.8%)
	Stage 2: 1 (2.8%)
	Stage 3: 20 (55.6%)
	Stage 4A: 2 (5.6%)
	Stage 4B: 2 (5.6%)
	Undetermined: 7 (19.3%)

^a^The severity of patients was determined by the highest stage of ROP in either eye

*FZD4* gene mutations were identified in four of the 36 ROP patients enrolled in our study, including three novel missense mutations (Table 2): p.A2P, p.L79M, and p.Y378C. No variants were found for *TSPAN12* and *NDP* genes in this study.

**Table 2:** Analysis of identified *FZD4* mutations in patients of our study

Patient ID	Exon	Nucleotide changes	Protein changes	Genotype	SIFT	Polyphen-2	Mutation Taster	ACMG	ExAC	gnomAD	Reference
P09	1	c.235C>A	p.L79M	Het	Damaging	PbD	DC	LP	NA	NA	Novel
P11											
P18	2	c.1133A>G	p.Y378C	Het	Damaging	PbD	DC	LP	0.00000824	0.00000657	Novel
P30	1	c.4G>C	p.A2P	Het	Damaging	Benign	Polymorphism	Uncertain	NA	NA	Novel

*Het* Heterozygous, *PbD* Probably damaging, *DC* Disease causing, *LP* Likely pathogenic, *NA* Not available

**Fig. 1:**
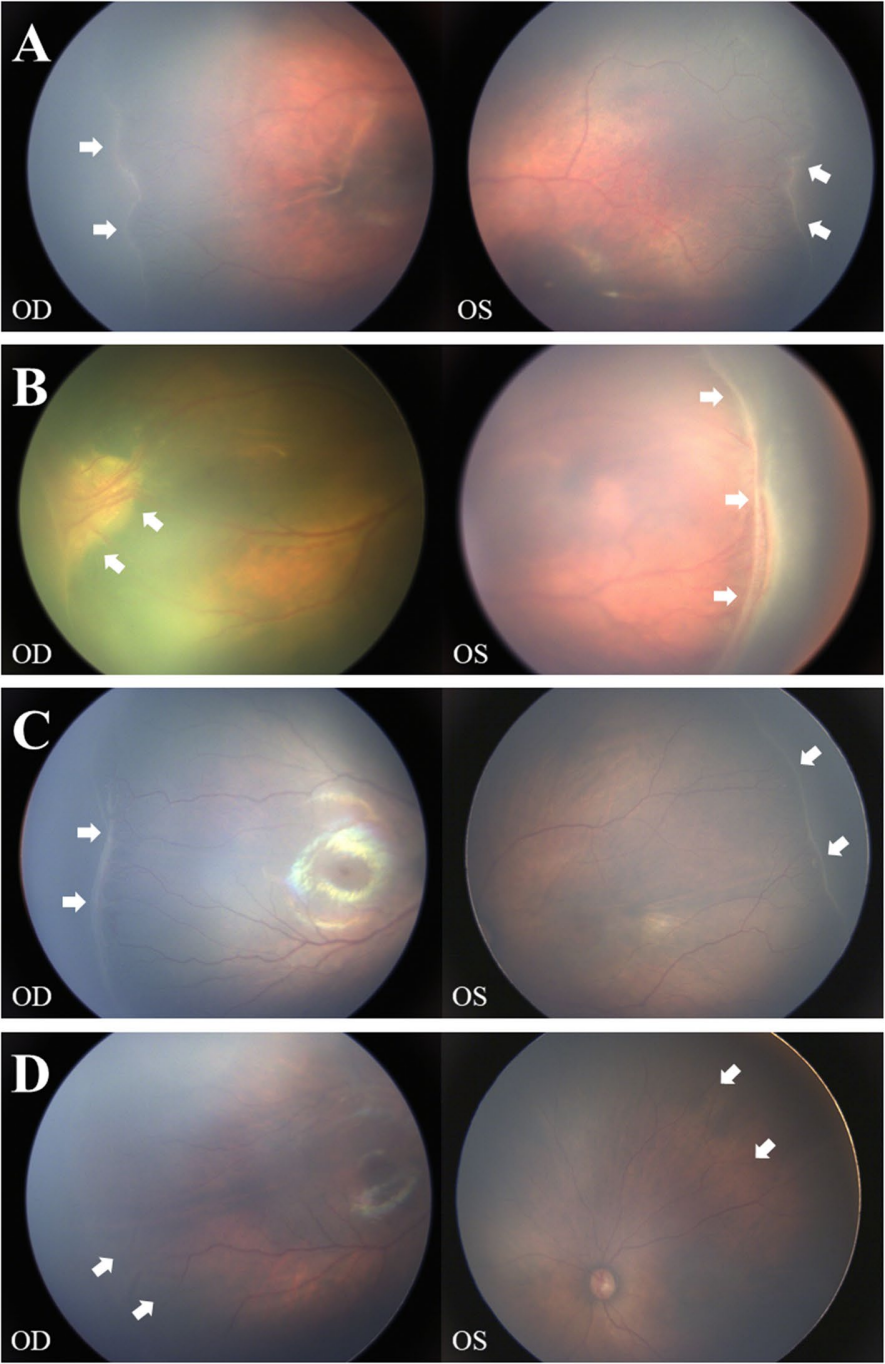
The color fundus photograph (CFP) of four ROP patients carrying mutations in *FZD4* gene. **a** The CFP of P09 demonstrates stage 3 of ROP, white arrows indicate the temporal irregular sprouts of vascularization at the ridge between vascular and avascular area. **b** The CFP of P11 demonstrates stage 4A of ROP, white arrows indicate the temporal partial retinal detachment. **c** The CFP of P18 demonstrates the temporal neovascular or proliferative alterations, which are related to stage 3 retinopathy and marked by white arrows. **d** The CFP of P30 demonstrates stage 0 of ROP, white arrows indicate the progressive tapering of retinal vessels

The clinical findings of the four patients who carried *FZD4* mutations are shown in Table 3. The variant p.L79M was detected in two patients with classic ROP. One patient was a boy (P09) who was born at a gestational age of 28 weeks with a birthweight of 1250 g; The retinopathy progressed to stage 3 in both eyes (Fig. 1a); Intravitreal ranibizumab (IVR) therapy was performed on both eyes at 10 weeks after birth. The other patient was a boy (P11) who was born at a gestational age of 33 weeks with a birth weight of 2400 g; The retinopathy developed to stage 4A bilaterally (Fig. 1b), and IVR treatment was performed on both eyes at 7 weeks after birth.

**Table 3:** Clinical characteristics of four ROP patients carrying *FZD4* mutations

Patient ID	Gender	Gestational age	Birth weight	Stage of ROP	Treatment
P09	Male	28 w	1250 g	3 (OU)	IVR (OU) at 10 w after birth
P11	Male	33 w	2400 g	4A (OU)	IVR (OU) at 7 w after birth
P18	Male	30 w	1150 g	3 (OU)	IVR (OU) at 9 w after birth
P30	Female	32 w	1950 g	0 (OU)	–

*w* weeks, *g* grams, *IVR* intravitreal ranibizumab

The p.Y378C mutation was found in one boy with classic ROP (P18). He was born at a gestational age of 30 weeks with a birthweight of 1150 g, and his fundus photography presented neovascular or proliferative alterations in both eyes (Fig. 1c), which are associated with stage 3 retinopathy; Bilateral IVR therapy was performed at 9 weeks after birth. The p.A2P mutation was detected in a girl (P30), she was born at a gestational age of 32 weeks with a birth weight of 1950 g, and her fundus photography showed immature retinal vascularization (stage 0) in both eyes (Fig. 1d).

**Fig. 2:**
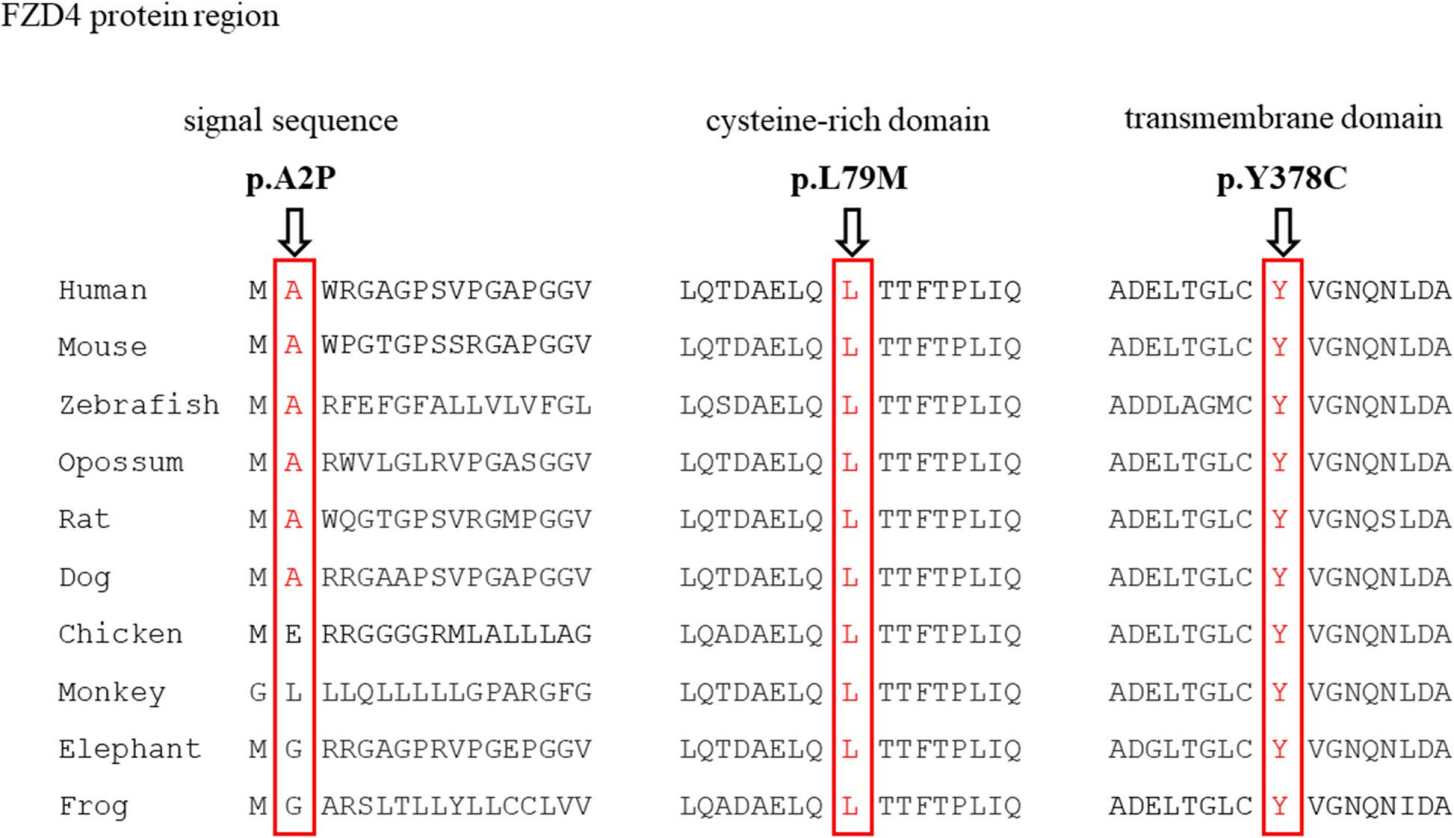
Sequence alignment of the FZD4 protein. Amino acids are shown by one letter code and red rectangles indicate the corresponding codons to *FZD4* variants found in our study, including codons A2, L79, and Y378

The two mutations p.L79M and p.Y378C are predicted to be damaging, probably damaging, and disease causing according to SIFT, PolyPhen-2, and Mutation Taster, and p.A2P is predicted to be damaging, benign, and polymorphism; In addition, p.A2P and p.L79M are never detected in ExAC and gnomAD, and p.Y378C is shown to be rare in the two databases (0.00000824 in ExAC, and 0.00000657 in gnomAD); According to the standards established by the ACMG for the classification of variants, p.L79M and p.Y378C are categorized as likely pathogenic, and p.A2P is classified as a variant with uncertain significance (Table 2). Additionally, Codon 2 is located in the signal sequence portion of *FZD4*, codon 79 lies upstream of cysteine-rich domain (CRD), and codon 378 is within the seven transmembrane domains (TMDs). Codon 79 and 378 are highly conserved in vertebrates, and codon 2 is conserved in all species except chicken, monkey, elephant and frog (Fig. 2). The CRD belongs to the extracellular region of FZD4 protein, and is found to be responsible for Norrin/β-catenin signaling pathway, as Norrin specifically binds to the CRD of FZD4 but not to CRDs of other 14 mammalian Frizzled or secreted Frizzled-related proteins [20], the mutational effects on CRD have been verified by altered cellular processing, plasma membrane targeting, interaction with Norrin ligand, and the ability to activate the signaling pathway [21]. The TMD is conserved among all the putative paralogs of the frizzled receptor family [22], and contains seven transmembrane α-helices [23]; The carboxyl-terminal region possesses a conserved K-T/S-XXX-W PDZ-binding motif that locates immediately after the hydrophobic TMD, this motif is necessary for canonical Wnt pathway initiation and Disheveled (Dvl) protein phosphorylation, and interaction of FZD4 with Dvl completely diminishes while the motif encountering any mutation [24]. The amino-terminal membrane localizing signal peptide sequence is rich in hydrophobic residues, and is known to translocate FZD4 to the plasma membrane, mutations that occur in this region might result in impaired stability of FZD4 protein and its mistranslocation subsequently [25].

## Discussion

ROP is known as a multifactorial disorder, gestational age, birth weight and supplemental oxygen are common risk factors. FEVR is an inherited retinal disorder characterized by incomplete retinal vascular development and secondary retinal changes, and has been reported to be associated with Wnt signaling genes (*FZD4*, *TSPAN12*, and *NDP*) polymorphism [26, 27]. As both diseases share similar clinical characteristics, the variants in these FEVR-causing genes are also considered to contribute to ROP pathogenesis. Here, our study showed that four of 36 ROP patients harbored three novel variants p.A2P, p.L79M, and p.Y378C in *FZD4* gene, no such sequence alterations were detected in *TSPAN12* and *NDP* genes.

*FZD4* is located on chromosome 11q14.2, and encodes a 537 amino acid protein, which belongs to the frizzled receptor family. It consists of a N-terminal extracellular cysteine-rich domain, seven transmembrane domains, extracellular/intracellular loops, and a C-terminal intracellular domain [28]. Frizzled-4 plays the role of receptor to bind specific ligands, which is essential for initiating different signaling pathways, such as canonical Wnt/β-catenin pathway, planar cell polarity pathway, and Wnt/Ca^2+^ pathway, this signaling pathway impairments could cause defective tissue homeostasis or cell proliferation in retina [29].

The p.L79M mutation in P09 and P11 replaces the non-polar leucine residue with the neutral and uncharged methionine residue; Furthermore, methionine is large, whereas leucine is small. This single-base substitution might cause an impact on CRD of *FZD4*, and lead to disrupted interaction between CRD and its ligands, further resulting in Wnt signaling dysfunction and retinal vascular alternations; All the structural or functional changes, combined with the absence of the *FZD4* variant in databases, indicate its pathogenicity in ROP. Furthermore, the gestational age and birth weight of P11 are relatively higher than that of other three patients with *FZD4* mutations, and his clinical features are similar to that of Li et al. [16], who reported seven atypical ROP patients presenting significantly greater gestational age and birth weight compared with typical ROP patients, so the phenotype of P11 might be attributed to the type of atypical ROP. It is noteworthy that P09 and P11 underwent different degrees of prematurity or other systemic abnormalities, the phenotypic severity in two patients carrying the same variant appeared to be inconsistent, suggesting that both genetic and environmental factors could affect the phenotypic heterogeneity in ROP.

In P18, variant p.Y378C is located in the seven transmembrane domains, tyrosine 378 belongs to the transmembrane ligand pocket of FZD4 [30], which is highly conserved in all species (Fig. 2); This mutation replaces the tyrosine residue with the smaller cysteine residue, it could impair the function of TMD and cause alterations in Wnt signaling pathway activation. Besides, the p.A2P mutated in P30 is in the signal sequence of *FZD4*, and could affect the process of FZD4 translocation to the plasma membrane; In this mutation, the non-polar alanine residue is replaced by a larger proline residue with the same properties, proline is an atypical amino acid with the characteristic of rigidity, which is crucial for the proper conformation of protein. Interestingly, P30 exhibited a milder phenotype compared with other three *FZD4*-mutated patients, this might be explained that the rigidity of proline did not affecting the important function of FZD4 protein completely, indicating the severity of mutational effect of *FZD4* gene could result in different ROP phenotypes. This finding is similar to that of Hiroyuki et al. [15], who suggested phenotypic severities being related to genotypic severities in patients with ROP.

## Conclusion

In this study, disease-causing mutations were not found in nearly 90% of participants in our study. No significant variants were detected in *TSPAN12* and *NDP* genes. Due to the limited number of ROP cases enrolled in this study, the correlation between phenotype and genotype in ROP patients was not entirely elucidated; In conclusion, our findings showed three novel missense mutations (p.A2P, p.L79M, and p.Y378C) of the *FZD4* gene, and broadened the mutational spectrum of ROP, implying the potential unique role of FZD4 in ROP pathogenesis; Although ROP seems to be a complex disorder involving both environmental and genetic factors, this study could provide useful information for better exploring the mechanism of this retinal vascular disease, thus highlighting the necessity of performing a comprehensive clinical and genetic screening to make progress in the diagnosis and treatment of ROP.

## Supplementary Information

**Additional file 1: Table S1.** Clinical findings of thirty-six patients with ROP.

## Data Availability

The data that support the findings of this study are available upon reasonable request from the corresponding author.
