# Supplementary material for: Ocular phenotype and genetical analysis in patients with retinopathy of prematurity
Source: BMC Ophthalmol. 2022 Jan 12;22:22. doi: 10.1186/s12886-022-02252-x (PMC8753894; doi:10.1186/s12886-022-02252-x)
Supplement: Supplementary file 1 — Additional file 1: Table S1. Clinical findings of thirty-six patients with ROP. [file 12886_2022_2252_MOESM1_ESM.docx]

**Table S1** Clinical findings of thirty-six patients with ROP.

| **Patient** | **Sex** | **GA (w)** | **BW (g)** | **Stage of ROP** | **Treatment** |
| --- | --- | --- | --- | --- | --- |
| P01* | F | 28 | 1150 | Undetermined | IVR (OU) |
| P02 | F | 29 | 1200 | 3 (OU) | IVR (OU) |
| P03 | F | 28 | 1220 | 3 (OU) | IVR (OU) |
| P04* | M | 29 | 1400 | Undetermined | LASER (OU) |
| P05 | M | 29 | 1400 | 4A (OD) | LASER (OD) |
| P06 | M | 28 | 1250 | 3 (OU) | IVC (OU) |
| P07 | M | 30 | 1480 | 3 (OU) | IVR (OU) |
| P08 | F | 29 | 1220 | 3 (OU) | IVR (OU) |
| P09 | M | 28 | 1250 | 3 (OU) | IVR (OU) |
| P10 | F | 30 | 1350 | 3 (OU) | IVR (OU) |
| P11 | M | 33 | 2400 | 4A (OU) | IVR (OU) |
| P12 | F | 28 | 1260 | 1 (OU) | – |
| P13 | M | 30 | 1350 | 4B/3 (OD/OS) | IVR (OU) |
| P14 | M | 28 | 1150 | 3 (OU) | IVR (OU) |
| P15* | M | 28 | 1350 | Undetermined | IVR (OU) |
| P16 | F | 29 | 1230 | 3 (OD) | LASER (OD) |
| P17 | M | 28 | 1350 | 3 (OU) | IVR (OU) |
| P18 | M | 30 | 1150 | 3 (OU) | IVR (OU) |
| P19 | M | 31 | 1050 | 3 (OU) | IVR (OU) |
| P20 | M | 28 | 1300 | 3 (OU) | IVC (OU) |
| P21 | F | 28 | 895 | 3 (OU) | LASER (OU) |
| P22 | M | 36 | 2700 | 0 (OU) | – |
| P23 | M | 34 | 2400 | 0 (OU) | – |
| P24 | F | 28 | 1350 | 3 (OU) | IVC (OU) |
| P25 | F | 28 | 900 | 3 (OU) | IVR (OU) |
| P26 | M | 29 | 1000 | 3 (OU) | LASER (OU) |
| P27 | M | 31 | 1140 | Undetermined | LASER (OU) |
| P28 | M | 32 | 1250 | Undetermined | NA |
| P29 | M | 30 | 1350 | 4B (OS) | NA |
| P30 | F | 32 | 1950 | 0 (OU) | – |
| P31 | F | 27 | 1100 | Undetermined | LASER (OU) |
| P32 | F | 28 | 970 | 2 (OU) | – |
| P33 | M | 29 | 890 | 3 (OD) | IVR (OD) |
| P34 | M | 30 | 1200 | 3 (OU) | LASER (OU) |
| P35 | M | 30 | 1250 | 3 (OU) | LASER (OU) |
| P36 | M | 29 | 900 | Undetermined | NA |

M, male; F, female; GA, gestational age; BW, birth weight; w, weeks; g, grams; IVR, intravitreal ranibizumab; IVC, intravitreal conbercept; NA, not available

*P01, P04 and P15 were classified as AR-ROP.
